# Breakthrough application of electrochromism: Multifunctional artificial muscle

**DOI:** 10.1016/j.isci.2024.109091

**Published:** 2024-02-06

**Authors:** Ling Wang, Bin Wang, Jingwei Chen, Haizeng Li

**Affiliations:** 1 College of Chemistry and Chemical Engineering, Dezhou University, Dezhou 253023, China; 2 Optics and Thermal Radiation Research Center, Institute of Frontier & Interdisciplinary Science, Shandong University, Qingdao 266237, China; 3 School of Materials Science and Engineering, Ocean University of China, Qingdao 266100, China; 4 Shenzhen Research Institute of Shandong University, Shenzhen, Guangdong 518057, China

**Keywords:** Physics, Engineering, materials science

## Abstract

In the article (Advanced Materials 2023; 2305914, https://doi.org/10.1002/adma.202305914) reported by Wang et al., electrochemically driven multifunctional electrochromic artificial muscles (EAMs) are demonstrated with intricate actuation and eye-catching color-change behaviors, which were assembled from V_2_O_5_ nanowires-carbon nanotube fibers-based high-twist electrochromic artificial muscle yarn (EAMY). With combined wet winding and wet twisting, excellent mechanical properties and uniform color change were achieved in the core-sheath EAMYs. Followed by hot pouring and molding of gel electrolyte, the contact problem between electrode and electrolyte was resolved and EAMs were fabricated with stable operation in the air, harvesting a high shrinkage stroke of 12% and a high reflectivity contrast of 51%. The judicious device architecture design and integration of multifunctionality will open new avenues for electrochromic technology.

## Highlights

• Electrochromic yarns of V_2_O_5_ nanowires-carbon nanotube fibers were obtained
• Multifunctional electrochromic artificial muscles were assembled with the yarns
• High shrinkage stroke (12%) and high reflectivity contrast (51%) were achieved

## Graphical abstract

**Figure:**
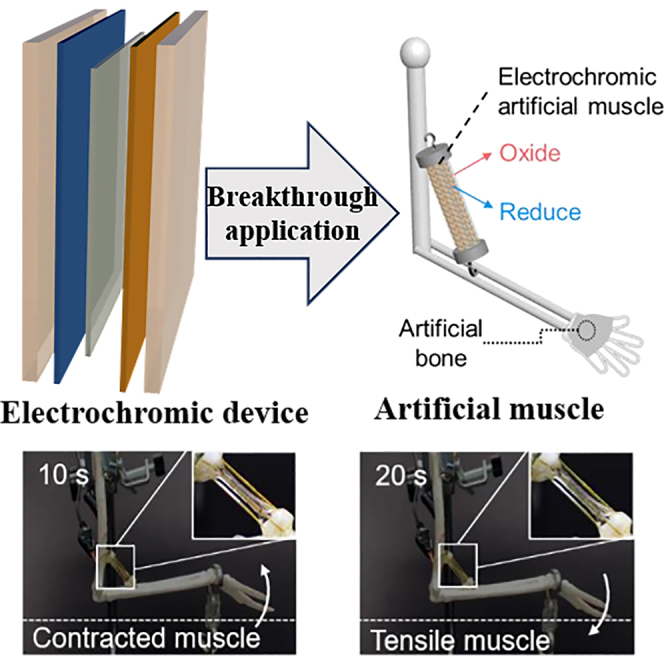

## Introduction

Electrochromism refers to the unique ability of certain materials, which can trigger reversible redox reactions under external electrical stimulation, thereby reversibly changing their color and/or optical properties (e.g., absorbance, transmittance, and reflectivity). These unique properties have been widely used in numerous areas such as the design of advanced structures, implantable medical devices, optical devices, and artificial electronic skins. As such, it has been an active area of basic and applied research. With the continuous development of science and technology, the demand for electrochromic devices is highly increasing, and the preparation of integrated high-performance multifunctional electrochromic devices has become a research hotspot. At present, multifunctional electrochromic energy storage devices,^1^^,^^2^ electrochromic sensors,^3^^,^^4^ electrochromic wearable devices,^5^^,^^6^ etc. have emerged in the field of multifunctional electrochromic devices.^7^ To achieve optimal performance with existing electrochromic materials and further enhance their applicability in practical environments, the bottlenecks of interfacial incompatibility between different layer of materials and how to achieve devices with multifunctionality are to be resolved.

## Results

Recently, Wang’s group reported multifunctional air-working electrochromic artificial muscles (EAMs) in *Advanced Materials* (https://doi.org/10.1002/adma.202305914).^8^ This is the first electrochemical artificial muscle that achieves both color-changing (reflectance contrast of 51%) and actuation functions (contractile stroke of approximately 12%) in the air. These results provide new possibilities for future application of EAMs in soft robots and artificial limbs.

**Figure 1:**
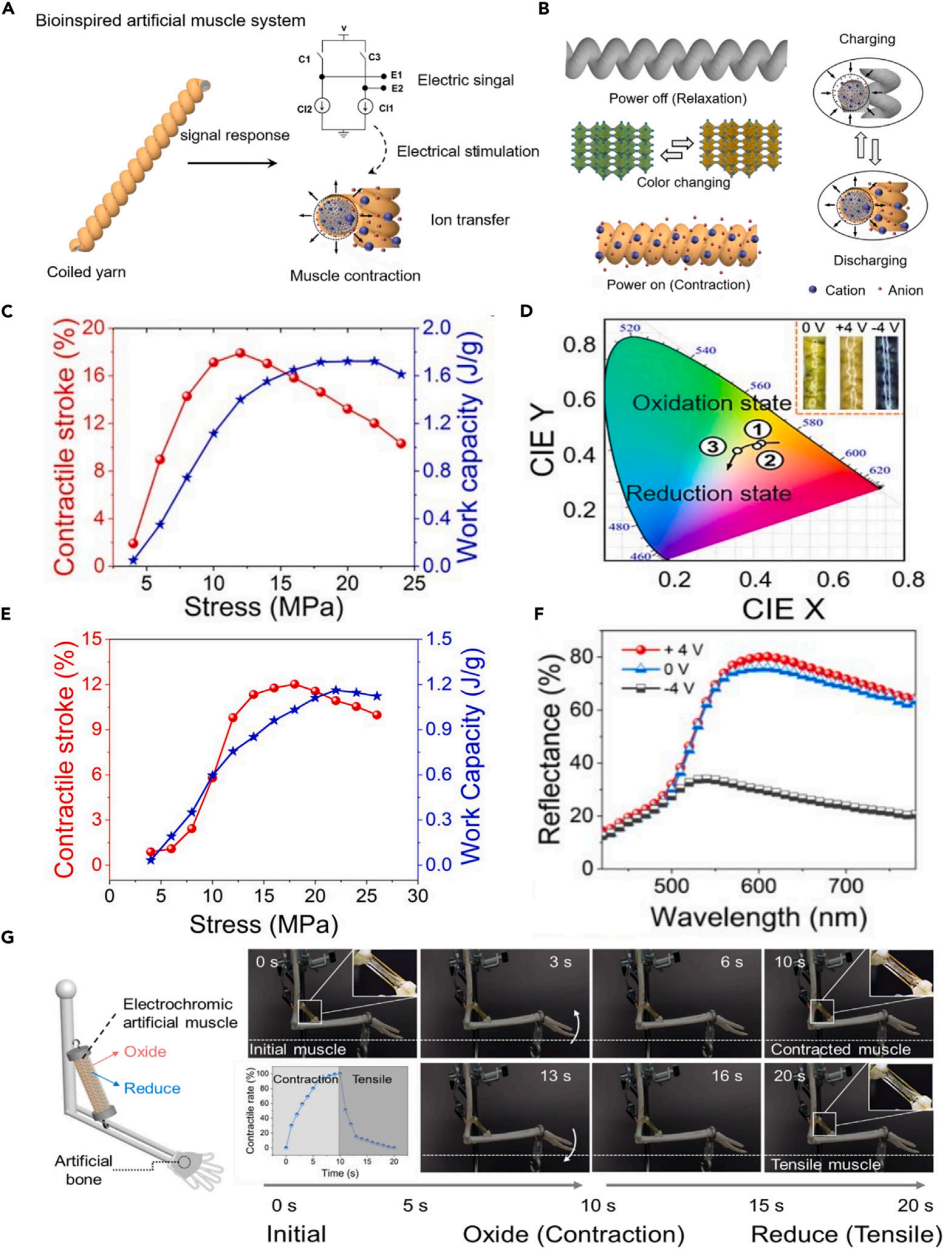
The EAMs based on V_2_O_5_ NWs-CNTF and its performance (A) Schematic of V_2_O_5_ NWs-CNT composite. (B) Schematic showing the actuation mechanism. (C) Contractile stroke and work capacity of EMAY. (D) The electrochromic properties of the EMAY. (E) Contractile stroke and work capacity of EAMs. (F) The electrochromic properties of the EAMs. (G) Reversible actuation and electrochromic of animal-tissue-like EAM. Reproduced with permission from ref. ^8^ Copyright 2023, Wiley-vCH.

Electrochemical artificial muscles have the advantages of breaking the Carnot limit of thermal muscles and sustain strokes at low input energies; meanwhile, electrochromism has excellent controllability and multicolor change characteristics under low-energy input. Combining the both advantages mentioned previously, electrochemically driven dual-response EAMs were constructed, which addressed the single actuation functionality of artificial muscles. Carbon nanotube fibers and multi-color-changing vanadium pentoxide nanowires were assembled into high-twist meter-scale electrochromic artificial muscle yarn (EAMY) with a tightly bound core-sheath layer by combining wet winding and wet twisting, providing stable bonding at the core-sheath interface, ensuring excellent mechanical properties and uniform color change (Figures 1A and 1B). Results revealed that core-sheath EAMY has good mechanical and electrochromic properties (Figures 1C and 1D). The EAMs were constructed by hot pouring followed by rapid cooling and molding of ionic liquid-based gel electrolyte layer and core-sheath EAMYs. The new electrolyte system and molding method have solved the contact problem between the electrolyte and the electrode during the actuation process, enabling stable operation in the air and further promoting the practicality of EAM. Through the new structure and manufacturing method mentioned previously, EAM couples the multifunctional color changing and actuation, achieving dual-response color-changing and actuation performance of EAM with a high shrinkage stroke of 12% (Figure 1E) and a high reflectivity contrast of 51% (Figure 1F) (at a sinusoidal input voltage of 4 V and a frequency of 0.05 Hz). Based on the aforementioned results, the multifunctional EAM could be installed at the elbow joint of an artificial human arm and actuated repeatedly to produce multiple color changes simultaneously (Figure 1G). As such, we believe that this multi-function integration strategy in the EAM can couple the intricate actuation and eye-catching color-change behaviors, and is expected to be applied in fields such as intelligent robots, wearables, and camouflage in the near future, further expanding the application fields of electrochromism.

## Discussion

This study is especially insightful and encouraging for the electrochromic, electrochemistry, and materials science community. Firstly, it combines electrochromic and electrochemical artificial muscles organically, achieving dual-responsive EAMs, inspiring researchers to explore new application of multifunctional electrochromic devices. Secondly, it shows that new assembly methods can effectively solve interface contact problems, maximize device performance, and lead to the optimal performance of EAM. Thirdly, the EAM’s stable operation in the air and excellent performance will inspire the development of next-generation multifunctional electrochemical and electronic devices as a challenging yet promising research direction.

To develop multifunctional electrochromic technology for practical applications, the following research directions can be identified: (1) low-cost synthetic routes for high-performance nanostructured electrochromic materials, (2) development of novel device architecture for emerging application scenarios, (3) integration of intelligent functions within the same devices, etc. It is recommended that the community closely pay attention to these issues and try to find ways to overcome these barriers. It is expected that multifunctional electrochromic technology will evolve from laboratory research to practical applications and commercial manufacturing in the foreseeable future.

## STAR★Methods

### Resource availability

#### Lead contact

Further information and requests should be directed to and will be fulfilled by the lead contact, Dr. Haizeng Li (haizeng@sdu.edu.cn).

#### Materials availability

This study did not generate new unique reagents.

#### Data and code availability

• All data reported in this paper will be shared by the lead contact upon request.
• This paper does not report original code.
• Any additional information required to reanalyze the data reported in this paper is available from the lead contact upon request.
